# Spatial Heterogeneity and Co-occurrence of Mucosal and Luminal Microbiome across Swine Intestinal Tract

**DOI:** 10.3389/fmicb.2018.00048

**Published:** 2018-01-26

**Authors:** Li Zhang, Weida Wu, Yuan-Kun Lee, Jingjing Xie, Hongfu Zhang

**Affiliations:** ^1^State Key Laboratory of Animal Nutrition, Institute of Animal Sciences, Chinese Academy of Agricultural Sciences, Beijing, China; ^2^Department of Microbiology and Immunology, National University of Singapore, Singapore, Singapore

**Keywords:** niches, 16s rDNA sequencing, microbiome, co-occurrence, bile acid, SCFA, pig

## Abstract

Pigs are one of the most important economic livestock. Gut microbiota is not only critical to the health but also the production efficiency of pigs. Manipulating gut microbiota relies on the full view of gut microbiome and the understanding of drive forces shaping microbial communities. 16s rDNA sequencing was used to profile microbiota along the longitudinal and radical axes to obtain the topographical map of microbiome in different intestinal compartments in young pigs. Alpha and beta-diversities revealed distinct differences in microbial compositions between the distal ileum and cecum and colon, as well as between the lumen and mucosa. *Firmicutes* and *Proteobacteria* dominated in the ileum, constituting 95 and 80% of the luminal and mucosa-attached microbiome. Transitioning from the small intestine to the large intestine, luminal *Bacteroidetes* increased from 1.69 to 45.98% in the cecum and 40.09% in the colon, while mucosal *Bacteroidetes* raised from 9 to 35.36% and 27.96%. Concurrently, luminal *Firmicutes* and *Proteobacteria* and mucosal-attached *Proteobacteria* remarkably decreased. By co-occurrence network analyses, *Prevotellaceae, Ruminococcaceae, Lachnospiraceae* and *Veillonellaceae* were recognized as the central nodes of luminal microbial network, and *Prevotellaceae* and *Enterobacteriaceae, Caulobacteraceae, Enterococcaceae, Xanthomonadaceae, Pseudomonadaceae* were identified as mucosal central nodes. Co-abundance was uncovered among *Prevotellaceae, Lachnospiraceae*, and *Veillonellaceae* in the luminal and mucosal microbiome, while opportunistic pathogens from γ-*Proteobacteria* in the mucosa. Strong co-exclusion was shown between *Enterobacteriaceae* with *Prevotellaceae*-centered microbial groups in the lumen. Redundancy analysis found bile acids and short chain fatty acids explained 37.1 and 41% of variations in the luminal microbial composition, respectively. Primary bile acid, taurine- and glycine- conjugated bile acids were positively correlated with *Lactobacillaceae, Enterobacteriaceae, Clostridiaceae_1, Peptostreptococcaceae*, whereas secondary bile acids, acetate, propionate, butyrate, and valerate were positively correlated with *Prevotellaceae, Acidaminococcaceae, Ruminococcaceae, Lachnospiraceae, Desulfovibronaceae, Veillonellaceae*. Functional analyses demonstrated that *Prevotella, Veillonellaceae, Lachnospiraceae*, and *Ruminococcaceae* were positively correlated with gene functions related to amino acids, energy, cofactors and vitamins metabolism, which are indispensable for the hosts. These results suggested site specific colonization and co-occurrence of swine gut microbiome closely relate to the microenvironment in each niche. Interactions of core gut microbiome greatly contributed to metabolism and/or immunity in the swine intestine.

## Introduction

Enormous numbers of microorganisms inhabit in the gastrointestinal (GI) tract, playing critical roles in gut maturation, nutrient digestion, vitamin synthesis, resistance to pathogens and immune modulation. Microbial community in the GI tract remains relatively stable once established, however, critical factors, such as lifestyle and antibiotics, can result in dramatic changes in the microbial community. Increasing evidence has shown that dysbiosis of gut microbial is associated with the pathogenesis of many inflammatory diseases and infections (Carding et al., 2015). For example, the use of antibiotics decreases the richness and diversity of gut microbiota, leading to subsequent antibiotic-associated diarrhea (Heinsen et al., 2015). Therefore, the assembly and structure of a microbial community are critical in maintaining homeostasis of the GI tract and the health of hosts. Until now, the mechanisms underlying the process of gut microbial community formation are still far from being fully understood.

Niches where the microbiota reside are considered as the deterministic drive to shape the gut microbial community. Availability of substrates, oxygen concentrations, pH and the interactions among microbes etc. together make up divergent niches in different compartments of the GI tract (Hollister et al., 2014; Pereira and Berry, 2017), which sustain distinct microbial communities (Stearns et al., 2011; Zhao et al., 2015). Along the length of the intestinal tract, a natural pH gradient rises from 6 in the duodenum to 7.4 in the terminal ileum of the human (Fallingborg, 1999). Under the enzymatic digestion from host, simple and easily-metabolizable carbohydrates are abundant and absorbed in the small intestine, and complex polysaccharides and shedding mucin accumulate in the large intestine (Walter and Ley, 2011). The richness in substrates and neutral pH enable the colonization and growth of a high load of microorganisms in the large intestine. The total population of bacteria is estimated to be ~10^11^–10^12^ CFU/ml in the large intestine compared with ~10^4^–10^8^ CFU/ml of contents in the small intestine (Rastall, 2004). Due to the pH-sensitivity, gram-positive *Firmicutes* dominated at mildly acidic pH levels, whereas *Bacteroides* outcompeted them at close to neutral pH levels (Duncan et al., 2009). The steep decreased concentrations of oxygen in the large intestine allows a high load of anaerobes, such as *Bacteroides* spp., *Clostridia* and other families within the *Clostridium* (including *Ruminococcus* spp., *Butyrovibrio* spp., *Fusobacterium* spp., *Eubacterium* spp., and *Peptostreptococcus*) (Rastall, 2004; Albenberg et al., 2014). Recently bile acids in the digestion fluid receive great interests regarding to their interactions with gut bacteria (Ridlon et al., 2014; Wahlström et al., 2016). Microbes possessing bile salt hydrolase (BSH) to metabolize bile acids, such as *Lactobacillus* and *Clostridium*, are more pronounced in the small intestine (Wahlström et al., 2016). Competition and cross-feeding between gut microbes further diversify the microbes in a specific niche. Mucolytic organisms provide mono- or oligosaccharides or partially-degraded mucins to bacteria without specialized mucolytic capability and enable the latter survives in the mucus layer of the intestine (Li et al., 2015). Considerable studies have been conducted to unravel links between intestinal microbial community with substrate availability, oxygen and pH. Additional factors in the intestine which shape the microenvironment are less investigated.

Pig is one of the most important economic livestock around the world. Investigation of gut microbiota is critical to maintenance health and production efficiency in pigs. Unraveling the processes and key components to shape the gut microbiome not only benefits the construction of a healthy gut of the animal but also provide important evidence for humans because pigs also serve as a biomedical model of human. Unlike tremendous studies have been done in humans and rodents in the past 10 years, the discovery of swine gut microbiome fell behind. Most of the available studies have focused on the effects of dietary intervention or antibiotics on the composition and structure of gut microbiota in pigs (Looft et al., 2014b; Sun et al., 2015, 2016; Umu et al., 2015; Kelly et al., 2016). Yang et al. (2016) and Looft et al. (2014a) have showed dramatic changes in the microbial composition and huge diversity among different sections of small intestine and cecum. Further steps are needed to figure out critical factors in shaping gut microbial community in pigs. In the present study, luminal and mucosa-associated microbiome were profiled along the swine intestine to obtain the taxonomic and functional shifts of microbiome and their co-occurrence network in different niches of pigs. Links between microenvironmental factors and microbiota were further established to understand critical drive forces to shape swine gut microbiome.

## Materials and methods

### Ethical approval

All animals were managed in accordance with the Animal Care and Use Committee of the Chinese Academy of Agricultural Sciences, compliant to the Regulations for the Administration of Affairs Concerning Experimental Animals (The State Science and Technology Commission of P. R. China, 1988).

### Animals and sample collection

Young Duroc × Landrace × Large White pigs (body weight: 11.05 ± 0.11 kg) were individually housed, allowed for free access to feed and water. Conventional corn-soybean meal diets were formulated according to Nutrient requirements of swine published by National Research Council in 2012, which provides 19.16% crude protein and 18.70 MJ/Kg gross energy to pigs weighing 11–15 Kg and 18.46% crude protein and 19.33 MJ/Kg gross energy to pigs weighing more than 15 Kg. No antibiotics was used before and during the experiment. Twenty-four pigs were sampled on two consecutive days. Each pig was sacrificed 2 h after the morning meal to facilitate luminal content collection. Sections of distal ileum, cecum and proximate colon were in situ ligated before the entire gastrointestinal tract was removed from the abdominal cavity. Mucosa and luminal contents from the middle segments of each gut section were sampled. Mucosa were first gently flushed with sterile phosphate-buffered saline and removed by scrapping with sterile glass microscope slides. Samples were immediately frozen in liquid nitrogen until DNA extraction and analysis of short chain fatty acids (SCFAs) and bile acids. Luminal contents were stored at −20°C prior to quantification of SCFAs.

### Quantification of SCFAs and bile acids

Luminal SCFAs, including acetate, propionate, butyrate, valerate, isobutyrate, and isovalerate were quantified using gas chromatograph (GC) as described by Wu et al. (2016). Short chain fatty acid in the digesta were extracted by distilled water. Metaphosphoric acid (25%, v/v) was added into the extracts at a ratio of 1:5 to remove protein. After centrifugation at 9,000 g, each supernatant was subjected for SCFA analysis with Agilent 6890N GC (Palo Alto, CA). Bile acids in the digesta were profiled using LC/MS/MS as described by Fang et al. (unpublished data). First, bile acids were extracted by sodium acetate buffer (pH 5.6, 50 mM) and ethanol in a water bath shaker at 37°C for 1 h. Extracts were collected by centrifuging at 20,000 × g for 15 min and passed through Bond Elute C18 cartridges (Harbor city, CA). Following a rinse with 20% ethanol, 5 ml of methanol was used to elute bile acids. Solvent were removed from the elutes by pressurized nitrogen gas. Each sample was reconstituted with 1 ml of methanol before subjecting to Waters Xevo TQ MS LC/MS/MS system. Standards for cholic acid (CA), chenodeoxycholic acid (CDCA), deoxycholic acid (DCA), ursodeoxycholic acid (UDCA), tauro-cholic acid (TCA), glyco-cholic acid (GCA), tauro-cholic acid (TCDCA), glyco-cholic acid (GCDCA), tauro-deoxycholic acid (TDCA), glyco-deoxycholic acid (GDCA), tauro-lithocholic acid (TLCA), tauro-ursodeoxycholic acid (TUDCA), glyco-ursodeoxycholic acid (GUDCA), tauro-hyodeoxycholic acid (THDCA) and chemical reagents were purchased from Sigma-Aldrich (Steinheim, Germany). Bile acids were categorized into primary bile acids (PBA, CA and CDCA), secondary bile acids (SBA, UDCA+DCA), taurine conjugated bile acids (TCBA) and glycine conjugated bile acids (GCBA).

### DNA extraction, amplification, and sequencing

Almost 0.5–1 gram (wet weight) of homogenized samples (144 samples) of luminal contents and mucosal tissues from ileum, cecum and colon of each pig was used. Genomic DNA was extracted using the manufacturer's protocol with the EZNA™ Soil DNA kit (D5625-02, Omega Bio-Tek Inc., Norcross, GA, USA). The quantity and quality of extracted DNA was measured using a NanoDrop2000 spectrophotometer (Thermo Fisher Scientific, Waltham, MA, USA) and agarose gel electrophoresis, respectively. Results showed that the A260: A280 ratios were 1.8–2.0, and that the DNA concentrations were between 10 and 500 ng/μL, indicating that the genomic DNA extracted met the requirements for subsequent sequencing. DNA samples were stored at −80°C prior to further analysis.

The V3–V4 hypervariable regions of the bacteria 16S rDNA were amplified by PCR using primers 338F (5′-ACTCCTRCGGGAGGCAGCAG-3′) and 806R (5′-GGACTACCVGGGTATCTAAT-3′) with unique 8-bp barcodes to facilitate multiplexing (Caporaso et al., 2012). 20 μL reactions were prepared containing 4 μL of 5 × FastPfu Buffer, 2 μL of 2.5 mM dNTPs, 0.8 μL of each primer (5 μM), 0.4 μL of FastPfu Polymerase and 10 ng of template DNA. The reactions were run on an GeneAmp® 9700 (Applied Biosystems, Foster City, CA, USA) with an initial denaturation at 95°C for 3 min; followed by 27 cycles of denaturation at 95°C for 30 s, annealing at 55°C for 30 s, extension at 72°C for 45 s; and a final extension at 72°C for 10 min. Amplicons were extracted from 2% agarose gels and purified using the AxyPrep DNA Gel Extraction Kit (Axygen Biosciences; Union City, CA, USA) according to the manufacturer's instructions and quantified using QuantiFluor™ -ST (Promega Corporation, Madison, WI, USA). The amplified, individually barcoded, 16S rDNA amplicons from each sample were pooled in equimolar and paired-end sequenced (2 × 250) on the Illumina MiSeq PE300 platform according to standard protocol (Caporaso et al., 2012). The raw sequence data were submitted to the NCBI SRA database (NCBI BioProject PRJNA402089).

### Quality-filtering and sequence analysis

Raw reads quality was strictly filtered using QIIME v1.9.0 (Quantitative Insights Into Microbial Ecology, http://qiime.org/index.html) software package with the following criteria (Bokulich et al., 2013): (i) the 250 bp reads were truncated at any site that obtained an average quality score <20 over a 50-bp sliding window, and the truncated reads shorter than 50 bp were discarded; (ii) reads with any mismatch in barcode, more than two nucleotide mismatches in the primer or containing ambiguous characters were removed; and (iii) overlapping sequences, shorter than 10 bp or with a mismatch ratio of more than 0.2 were eliminated.

The remaining high-quality sequences were clustered into operational taxonomic units (OTUs) at 97% sequence identity using UPARSE (version 7.1, http://drive5.com/uparse/) pipeline (Edgar, 2013) and the chimera sequences arising from the PCR amplification were detected and excluded from the OTUs using UCHIME (version 4.2.40) (Edgar et al., 2011). Taxonomic assignment of OTUs was performed with the mother Bayesian classifier (70% confidence) with the MOTHUR formatted version of the Ribosomal Database Project (RDP, version 11.1, http://rdp.cme.msu.edu/) (Maidak et al., 2001).

### Analyses of α- and β- diversity

Alpha-diversity, Chao1, ACE, Shannon, and Simpson's evenness indexes were calculated at 97% identity (Paul et al., 2015) and plotted using the “phyloseq” package (McMurdie and Holmes, 2013). Rarefaction metrics were computed using the alpha_rarefaction.py script in the Qiime package and plotted using R programme (v3.3.0, https://www.r-project.org/). Each library size was rarefied at the lowest sequence reads of all investigated samples. Beta-Diversity was investigated with QIIME using principal coordinated analysis (PCoA) based on weighted (assessment of community structure by considering the abundance of OTU) and unweighted (assessment of community membership by considering the presence/absence of OTU) UniFrac distance matrix (Lozupone and Knight, 2005). Analysis of similarities (ANOSIM) was performed to assess the overall similarity among intestinal niches by testing the significance of spatial separation in PCoA. *R*-value of ANOSIM indicated distinct microbiota to similar microbiota from 1 to 0 (*R* > 0.75, good separation; *R* > 0.5, overlapping; *R* < 0.25, no separation) (Clarke, 1993). PICRUSt (v1.0) was used to predict microbial gene functions against KEGG pathway database based upon 16S rDNA sequencing data (Langille et al., 2013). Differences in predicted gene functions of bacterial communities among luminal or mucosal samples were determined by principal component analysis (PCA) using the SIMCA-P (v11.5) software package (Umetrics; Umea, Sweden). OTUs that appeared in at least 50% samples per examined niche with a minimum count of 100 sequences per OTU were selected to generate core microbiota in luminal or mucosal samples. Correlations between core OTUs and microbial gene functions were estimated by a Spearman correlation coefficient and selected examples were visualized in a heatmap generated by R using the “microbiome” package (URL: http://microbiome.github.com/microbiome).

### Co-occurrence analysis of core microbiome

SparCC (Sparse Correlations for Compositional data) was employed to determine co-abundance (positive) and co-exclusion (negative) relationships between bacteria taxa at the absolute abundances (Friedman and Alm, 2012). SparCC and calculation of two-side pseudo *p*-values were run on python scripts based on bootstrapping of 100 repetitions. A network plot was generated for luminal and mucosal microbiota, respectively, and correlation magnitudes over 0.6 were plotted when pseudo *P*-value was <0.05.

### Redundancy analysis (RDA)

Relationship between luminal microbial composition and bile acids and SCFAs was determined with RDA which was implemented in the Canoco 5.0 software package (Microcomputer Power, Ithaca, NY). Bile acids and SCFAs composition were introduced as explanatory variables. The relative contributions of the 28 family-level (average relative abundance >1% in at least one region) phylogenetic groups were used as response variables. The Monte Carlo Permutation test (*N* = 499) with a *P* < 0.05 indicates a significant relationship between bile acids and SCFAs and the microbial composition.

### Statistical analysis

Nonparametric Mann-Whitney (MW) or Kruskal-Wallis (KW) tests were employed to test the treatment effects of two groups or more, respectively, and *post-hoc* Dunn-Bonferroni tests were performed for pairwise comparisons using SPSS for Windows (v.21, Chicago, IL).

## Results

### Bile acids and SCFAs in different intestinal compartments

**Figure 1:**
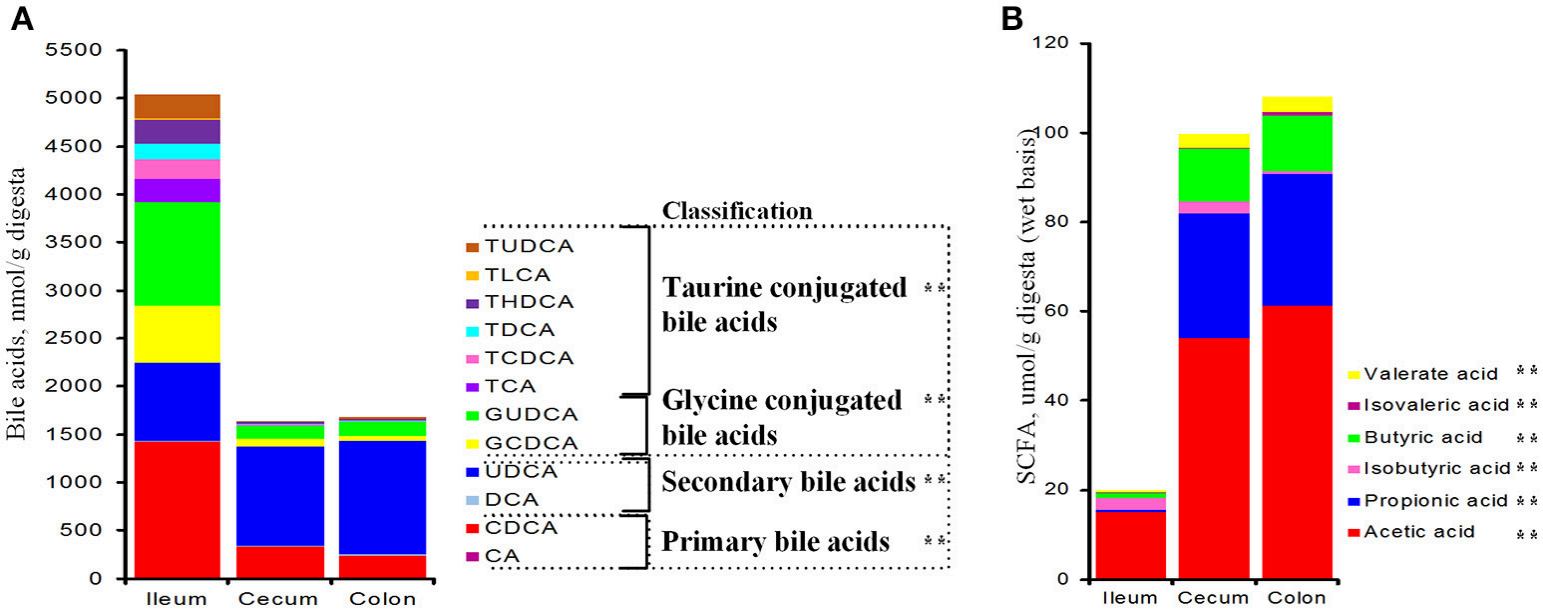
Bile acids **(A)** and short chain fatty acids **(B)** in the distal ileum, cecum and proximate colon of growing pigs. Values are expressed as means. Items with two asterisks represents a significant difference (*P* < 0.001). BA, bile acid; CA, cholic acid; CDCA, chenodeoxycholic acid; DCA, deoxycholic acid; LCA, lithocholic acid; UDCA, ursodeoxycholic acid; HDCA, hyodeoxycholic acid; TCA, tauro-cholic acid; GCA, glyco-cholic acid; TCDCA, tauro-cholic acid; GCDCA, glyco-cholic acid; TDCA, tauro-deoxycholic acid; GDCA, glyco-deoxycholic acid; TLCA, tauro-lithocholic acid; TUDCA, tauro-ursodeoxycholic acid; GUDCA, glyco-ursodeoxycholic acid; THDCA, tauro-hyodeoxycholic acid.

Total bile acids in the ileum were 3-fold as much as those of the cecum and colon (*P* < 0.001, Figure 1A). Ileal PBA was about 430 and 580% greater than those of the cecal and colonic sections, respectively (*P* < 0.001); whereas SBA was about 25% less (*P* = 0.002, Figure 1A). Over 55% ileal bile acids were conjugated with taurine or glycine and the ratio dropped below 17% in the cecum and colon. From the ileum to the large intestine, total amount of conjugated bile acids was significantly reduced by 90% (*P* < 0.001), while free bile acids (PBA + SBA) were lower by 40% (*P* < 0.001, Figure 1A). Both glycine- and taurine-conjugated bile acids were lower in the cecum and colon than the ileum (*P* < 0.001, Figure 1A). In general, the composition of PBA, SBA, GCBA and TCBA was 30:15:35:20 in the ileum, 20:60:15:5 in the cecum and 15:70:10:5 in the colon. Total concentration of SCFAs in the large intestine was 5-fold higher than that of the ileum (*P* < 0.001, Figure 1B) and each SCFA, including acetate, propionate, butyrate, isobutyrate, valerate and isovalerate, was significantly greater.

### Global sequencing data

A total of 5,935,104 valid sequences were obtained from 144 intestinal luminal and mucosal samples (*n* = 24), with an average of 41,216 sequences per sample. After data trimming and quality filtering, 5,001,571 high-quality sequences (representing ~84% of the total sequences) were acquired, with an average of 34,733 sequences per sample (ranging from 25,655 to 43,561). Downstream analyses described in the results are based on the normalized depth of 25,648 reads per sample to account for differences in sequencing depths (Figure S1). The high-quality sequences, 3,693,312, clustered into 2,365 operational taxonomic units (OTUs; 97% identity), representing independent species belonging to 726 genera, 318 families, 173 orders, 93 classes, and 45 phyla. Results showed that the all Good's coverage was >0.99, implying that most of microbial diversity within the luminal and mucosal samples had been sufficiently captured.

### Spatial changes in luminal and mucosal microbiota composition

**Figure 2:**
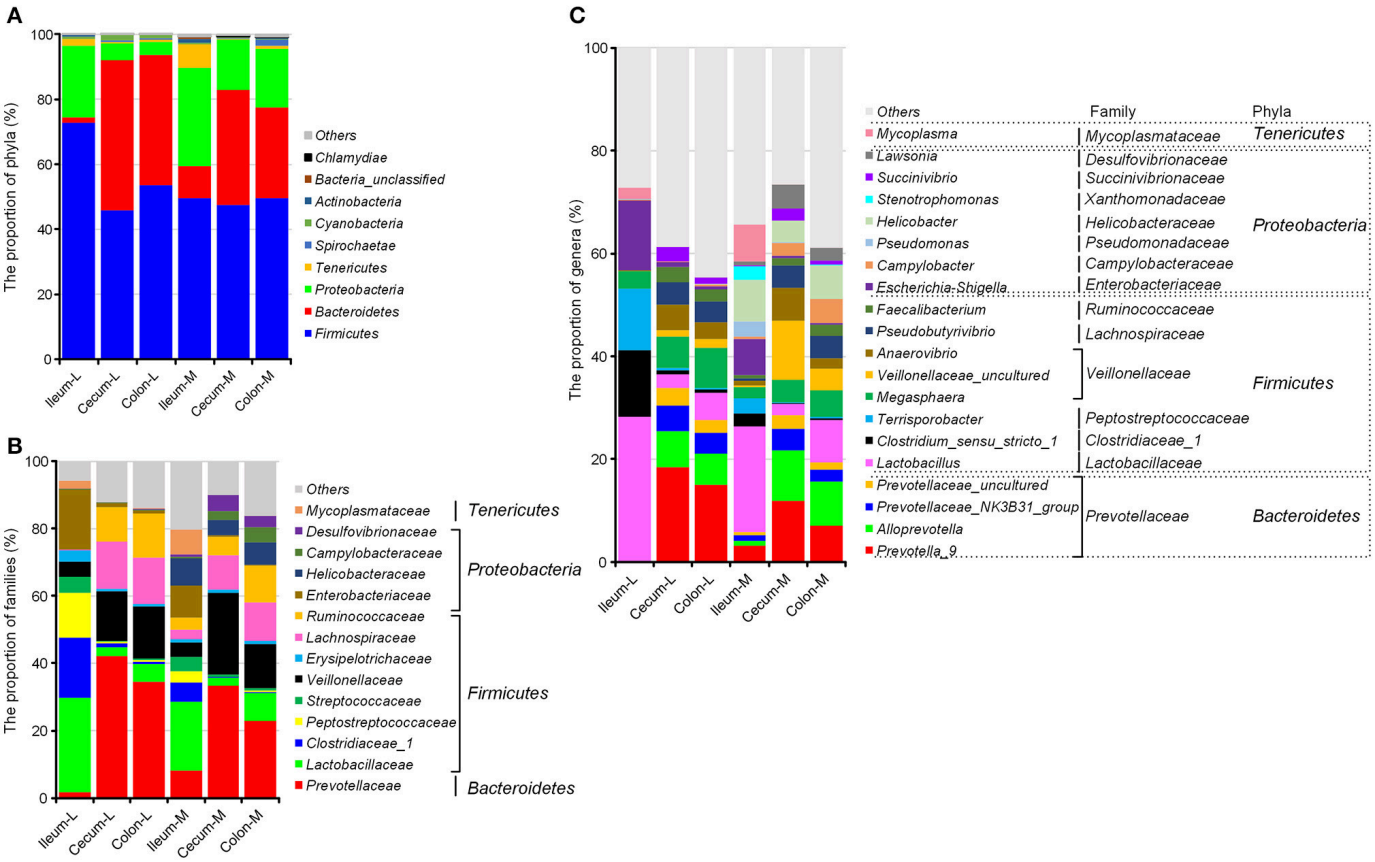
Spatial distribution of the most abundant taxa of intestinal microbiota in the distal ileum, cecum and proximate colon of growing pigs at the level of phylum **(A)**, family **(B)**, and genus **(C)**. Values are expressed as means. Only taxa that occupied more than 0.5% in at least one region at the phylum level or more than 3% at the family and genus level are presented.

The greatest number of bacterial phyla with a relative abundance ≥ 0.05% was identified in the microbiota attached to the ileal mucosa among all six niches, where *Gemmatimonadetes* and *Planctomycetes* were exclusively found (Table S1). In general, microbiota in swine intestinal niches was dominated by *Firmicutes, Proteobacteria, Bacteroidetes, Actinobacteria*, and *Cyanobacteria*. Microbial composition changed greatly from the ileum to the large intestine. In the ileum, *Firmicutes* (72.82%) and *Proteobacteria* (21.92%) made up 95% of luminal microbiota, and the proportion of these two phyla were reduced to 51.08 and 57.58% in the cecum and colon, respectively (*P* < 0.001). Meanwhile, *Bacteroidetes* was highly increased from 1.69% in the ileal lumen to 45.98 and 40.09% in the two sections of the large intestine (Figure 2A, Table S1). Mucosa-attached *Firmicutes* was at a relatively stable level close to 50% across all three intestinal sections, whereas *Bacteroidetes* (9.08% in the ileum) was increased by 3.6 and 2.85-fold in the cecal (35.36%) and colonic mucosa (27.96%), but not as profound as in the lumen. Mucosal *Proteobacteria* was greater than the luminal counterparts and its proportion went down from 30.13% in the ileum to 15.45 and 18.04% in the cecum and colon, respectively. In the ileum, mucosal microbiota was less abundant in the *Firmicutes* but greater in *Proteobacteria* and *Bacteroidetes*. In contrast, major compositional differences between mucosal and luminal microbiota in the colon and cecum were the increased *Proteobacteria* and the decreased *Bacteroidetes* in the mucosa (Figure 2A).

At the family level (Figure 2B, Table S2), greater *Prevotellaceae* within *Bacteroidetes* while less *Enterobacteriaceae, Campylobacteraceae, Helicobacteraceae*, and *Desulfovibronaceae* within *Proteobacteria* were found in the large intestine than the ileal sections. Although the proportion of the *Firmicutes* phylum was significantly reduced in the luminal microbiota colonized in the cecum and colon, *Veillonellaceae, Lachnospiraceae* and *Ruminococcaceae* within *Firmicutes* were increased when *Lactobacillaceae* was decreased in the mucosa and lumen of the large intestinal sections.

*Campylobacteraceae, Helicobacteraceae* and *Desulfovibronaceae* within *Proteobacteria* were almost exclusively present in the mucosa-attached microbiota.

At the genus level, *Lactobacillus, Clostridium_sensu_stricto_1, Tenisporobacter* and *Escherichia*-*Shigella* dominated the ileal microbiota and significantly decreased in the large intestine (Figure 2C, Table S3). Five genera of *Prevotellaceae* (*Prevotella-9, Alloprevotella, Prevotellaceae_NK3B31_group, Prevotellaceae_uncultured, Prevotella_2*), two genera of *Veillonellaceae* (*Megasphaera* and *Veillonellaceae_uncultured*) and *Faecalibacterium* of *Ruminococcaceae* were greater in the cecum and colon in comparison with the ileal section. Comparisons between luminal and mucosa-attached microbiota revealed that *Campylobacter, Helicobacter, Pseudomonas*, and *Lawsonia* within *Proteobacteria* were increased, whereas *Prevotella_9 and Prevotella 2* within *Prevotellaceae* were decreased in the mucosa of cecum and colon.

### Diversity and richness of luminal and mucosal microbiota

**Figure 3:**
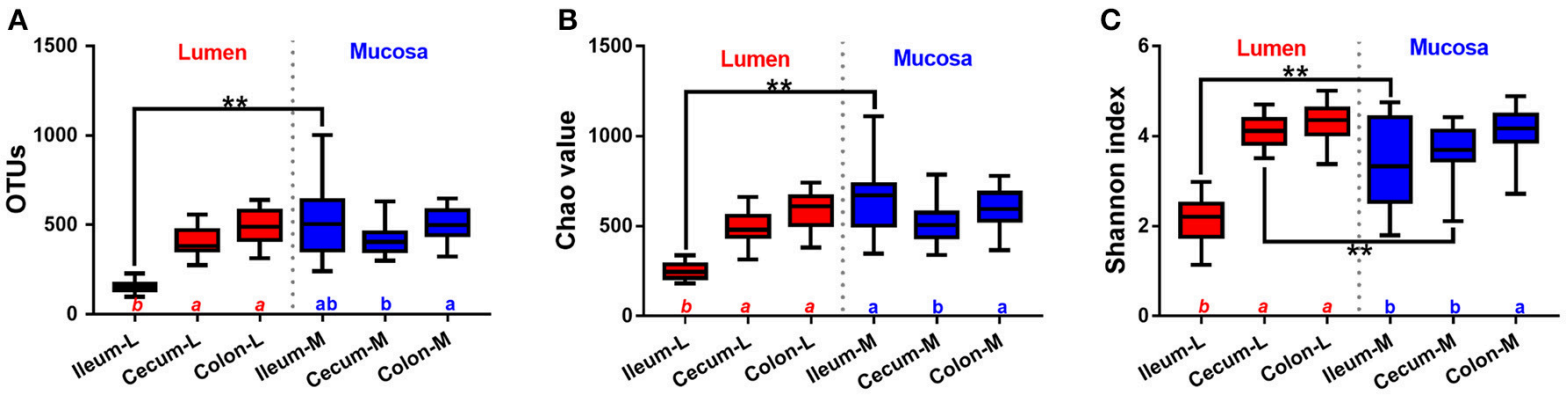
Alpha-diversity of microbiome residing in the lumen and mucosa of the distal ileum, cecum and proximal colon of pigs. **(A)**, OTUs; **(B)**, Chao value; **(C)**, Shannon index. Values are expressed as means ± standard error. Boxes with an asterisk symbol above their whiskers are significantly different between the lumen and the corresponding mucosa in each intestinal niche. Boxes with a different letter (within the same color) under their whiskers are significantly different among the luminal samples (red) as well as other mucosal samples (blue).

Numbers of OTUs, Chao 1 and Shannon index, indicating α-diversity of microbiota, showed significant differences among intestinal niches (Figure 3). Among luminal samples, α-diversity parameters were substantially increased in the cecum and colon compared with ileum. In contrast, less intra-segment variations were observed in mucosa-attached microbiota, where only slight differences were found in the number of OTUs, Chao 1 and Shannon index between the cecum and colon. Of note, the total number of OTUs identified in the microbiota attached to the ileal mucosa was almost 3.5 times that identified in the luminal samples (Figure 3A). Compared with luminal microbiota, Chao 1 (Figure 3B) and Shannon index (Figure 3C) were smaller for bacteria attached to mucosa in the ileum and cecum.

**Figure 4:**
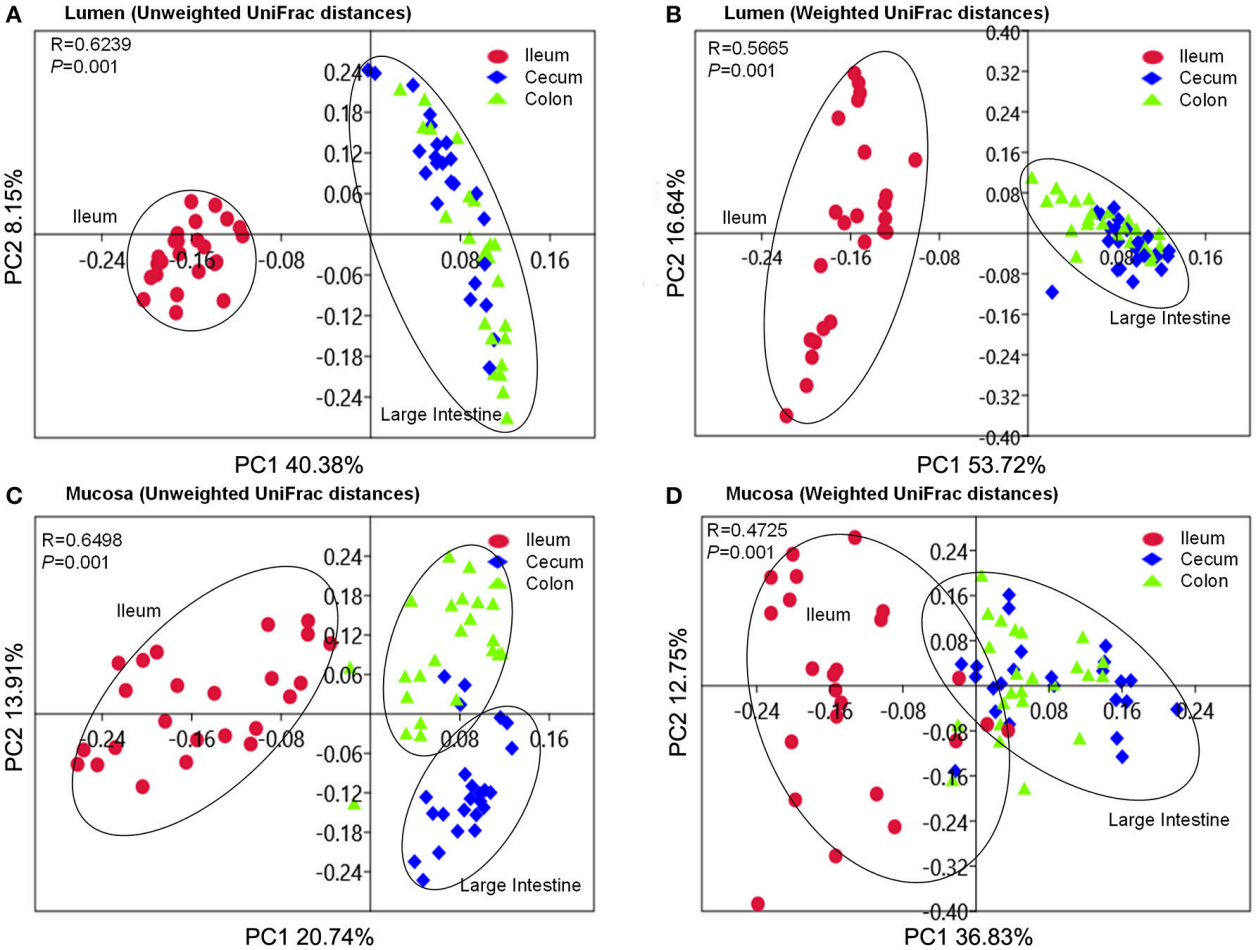
PCoA plots based on unweighted and weighted UniFrac distances of luminal microbiome **(A,B)** as well as mucosa-attached microbiome **(C,D)**. Respective ANOSIM R values showing the extent of community variation among different intestinal segments and statistical significance were indicated. Axes represent the two dimensions explaining the greatest proportion of variances in the communities for each analysis.

Beta-diversity was assessed by the unweighted and weighted principal coordinate analysis (PCoA). It was clear that the ileal microbiota either attached to the mucosa or colonized in lumen clustered together to separate from the cecal and colonic microbiota (Figure 4). Cecal microbiota did not separate from the colonic community except those attached to the mucosa in the unweighted PCoA plot (Figure 4C). ANOSIMs also confirmed the structural dissimilarity between the ileal microbiota and counterparts colonized in the large intestine. Luminal microbiota from ileum was well separated from those in the large intestinal sections (*R* > 0.75, Table 1); whereas microbiota attached to mucosa overlapped a little with those that colonize the lumen of cecum and colon (0.5 < *R* < 0.75, Table 1). Similar structure of luminal microbiota community was found in the cecum and colon as *R*-values were below 0.25 when an overlap in community structure was indicated in the cecal and colonic bacteria attached to mucosa. Using unweighted UniFrac distance, mucosa-attached microbiota showed significant dissimilarities with the luminal microbiota in the ileum and cecum. In contrast, only modest separations were observed between the mucosal and luminal microbiota in all three intestinal sections using the abundance-weighted model (Table 1).

**Table 1:** ANOSIM *R*-values based on weighted and unweighted UniFrac distances of microbial community between variables (Sample size *n* = 24, Number of permutation tests are 999).

**Variables**	**Unweighted R^a^**	***P*-value**	**Weighted R^a^**	***P*-value**
All niches	0.6132	0.001	0.5096	0.001
**LUMEN VS. MUCOSA**				
Ileum	0.8471	0.001	0.2621	0.001
Cecum	0.6581	0.001	0.2905	0.001
Colon	0.1851	0.001	0.2662	0.001
**LUMEN**				
Ileum vs. Cecum	0.9925	0.001	0.9032	0.001
Ileum vs. Colon	0.9979	0.001	0.8691	0.001
Cecum vs. Colon	0.1936	0.002	0.0752	0.019
**MUCOSA**				
Ileum vs. Cecum	0.7279	0.001	0.6493	0.001
Ileum vs. Colon	0.5883	0.001	0.664	0.001
Cecum vs. Colon	0.7163	0.001	0.1754	0.002

### Co-occurrence of core microbiota in lumen and mucosa

**Figure 5:**
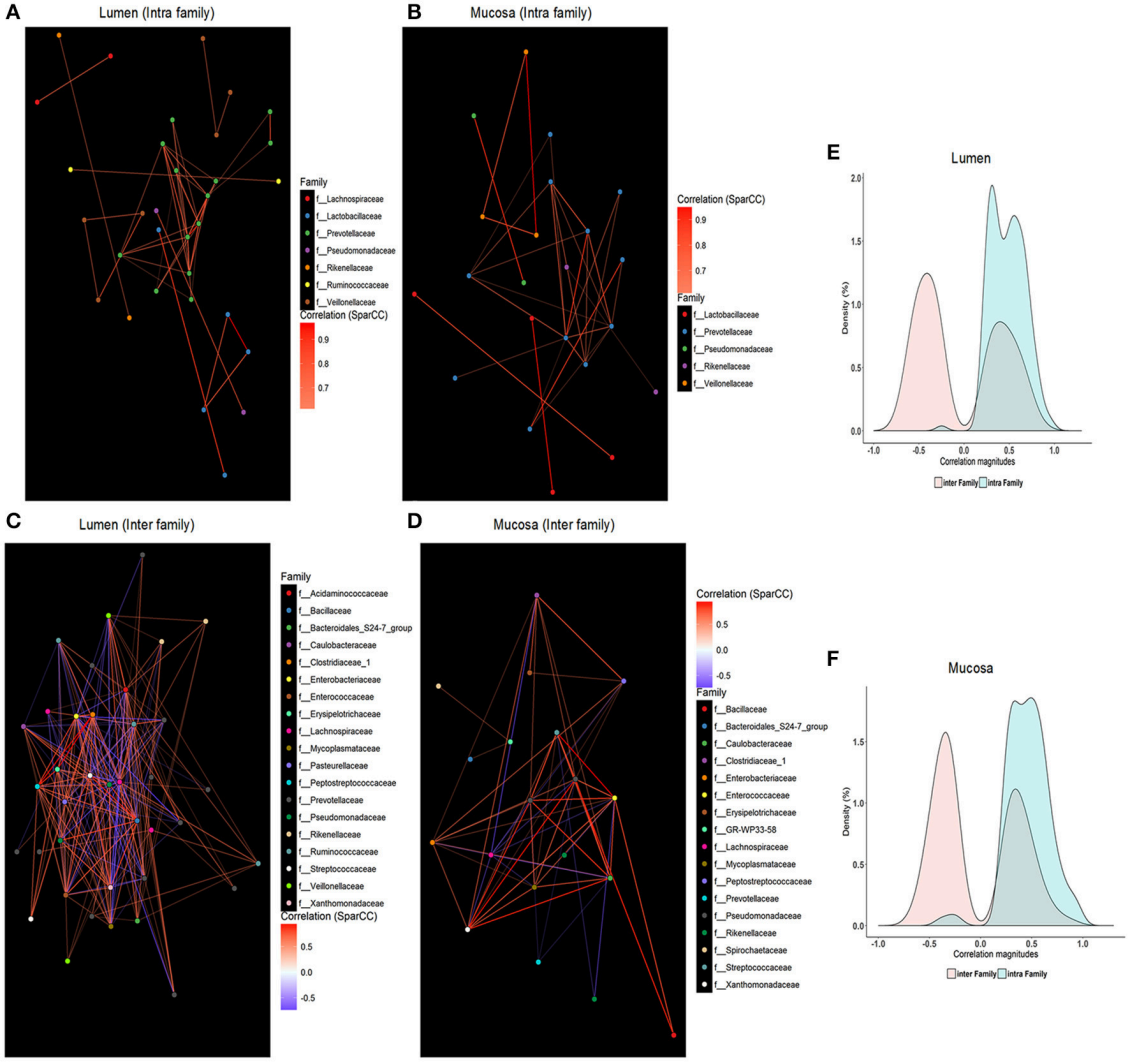
Intra-family and inter-family networks between core OTUs were constructed for luminal samples **(A,C)** and mucosal samples **(B,D)**, respectively. The color of each node indicates the taxonomic group of those core OTUs at the family level. Only significant correlations (two-sided pseudo *p* ≤ 0.05 based on bootstrapping of 100 repetitions) with an absolute correlation magnitude ≥ 0.6 were presented. Nodes represent OTUs involved in either significant co-abundance (red edges) or co-exclusion (blue edges) relationships. Density plots indicate the magnitudes of the significant inter-family and intra-family correlation (*p* ≤ 0.05 following bootstrapping) among core OTUs in the luminal microbiome **(E)** and the mucosa-attached microbiome **(F)**.

SparCC was employed to characterize co-occurrence relationships between 64 core OTUs, and 1,550 and 1,099 significant correlations were revealed in the microbiome residing in the lumen (Table S4) and mucosa (Table S5), respectively. When within-site relationships were considered, the ratio of co-abundance to co-exclusion (787 vs. 763, 556 vs. 543) was close to 1 in the lumen or mucosa, which is similar to findings of Faust et al. (2012). Interestingly, almost all intra-family correlations in luminal (Figure 5E) or mucosal (Figure 5F) microbiota were positive, whereas negative edges were slightly greater than positive ones in the inter-family relationships (lumen, 618 vs. 762; mucosa, 432 vs. 540), respectively. Co-occurrence networks were visualized with core OTUs of strong correlations (i.e., SparCC correlation magnitude of ≥0.6, *p* ≤ 0.05, Figure 5). Families within *Firmicutes* and *Bacteroidetes* were mainly found as the hubs of luminal microbiota, whereas the main hubs of mucosal microbiota were families from *Proteobacteria* and *Bacteroidetes* phyla. Core OTUs of *Prevotellaceae* belonging to *Bacteroidetes* were the central nodes for both luminal and mucosal microbiota and established extensive correlations with other OTUs, which accounted for almost 25% of total correlations. In addition to *Prevotellaceae, Ruminococcaceae, Lachnospiraceae, Veillonellaceae*, and *Streptococcaceae* within *Firmicutes* were the most correlated hubs for the luminal microbiota, whereas *Pseudomonadaceae, Enterococcaceae, Caulobacteraceae, Xanthomonadaceae*, and *Enterobacteriaceae* within *Proteobacteria* and *Lachnospiraceae* within *Firmicutes* were the main hubs for mucosal microbiota (Table S6).

Compared with mucosa-attached microbiota, a greater number of inter-family correlations (Figures 5C,D), but comparable intra-family correlations, were revealed in the luminal microbiota (Figures 5A,B). Positive correlations were identified among OTUs of *Prevotellaceae, Veillonellaceae, Lactobacillaceae*, and *Pseudomonadaceae* in both luminal (Figure 5A) and mucosal microbiota (Figure 5B). However, correlations between OTUs of *Ruminococcaceae* and *Lachnospiraceae* were only apparent in luminal samples (Figure 5C). OTUs of *Enterobacteriaceae* were negatively correlated with those of *Prevotellaceae, Ruminococcacea*e, and *Lachnospiraceae* and *Veillonellaceae* in the lumen (Figure 5C) but positively correlated with those of *Caulobacteraceae, Enterococcaceae, Xanthomonadaceae*, and *Pseudomonadaceae* in the mucosa (Figure 5D).

### Microbiome responding to luminal bile acids and SCFAs in the different gut sections

**Figure 6:**
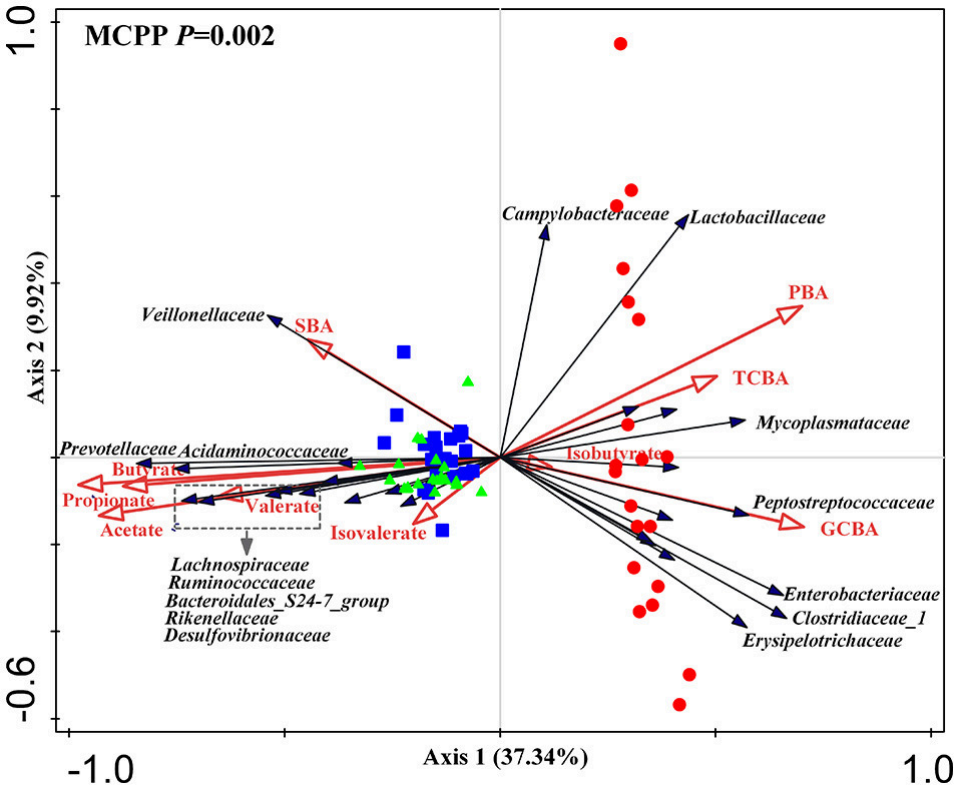
Triplot of redundancy analysis (RDA) of the intestinal microbial composition at the family level relative to luminal bile acids and SCFAs. Only taxa with an average relative abundance ≥ 1% in at least one region were involved. Microbiota from the distal ileum, cecum and colon were indicated by red circles, blue square and green triangles, respectively. Constrained explanatory variables (primary bile acid, PBA; secondary bile acid, SBA; taurine-conjugated bile acid, TCBA; glycine-conjugated bile acid, GCBA; SCFAs including acetate, propionate, butyrate, valerate, isobutyrate and isovalerate) were indicated by red arrows. Responding taxa were indicated by black arrows and only those with highest fit in ordination plot were labeled. First and second coordinates were plotted, showing 37.34 and 9.92% of the total variability in the data set, respectively. Top-left, *P*-value was obtained by Monte Carlo permutation procedure (MCPP).

The difference observed in redundancy analysis was significant (*P* = 0.002) as assessed by Monte Carlo Permutation Procedure. In total, 51.6% of total variations in microbiota composition at the family level were related to luminal bile acids and SCFAs. Among them, luminal bile acids explained 37.1% of the total variations and SCFAs did 41%. The first and second principal component contributed to 37.34 and 9.92% of total variations (Figure 6). The triplot of RDA showed that the ileum and large intestine samples were separated at the first constrained axis. *Lactobacillaceae, Enterobacteriaceae, Clostridiaceae_1, Peptostreptococcaceae, Mycoplasmataceae, Campylobacteraceae* and *Erysipelotrichaceae* were positively correlated with PBA, GCBA, and TCBA, while *Prevotellaceae, Acidaminococcaceae, Ruminococcaceae, Lachnospiraceae, Desulfovibronaceae, Veillonellaceae, Bacteroidales_S24.7_group, Rikenellaceae* were positively correlated with SBA and acetate, propionate, butyrate and valerate.

### Predicted gene functions of microbiota that colonize different niches

**Figure 7:**
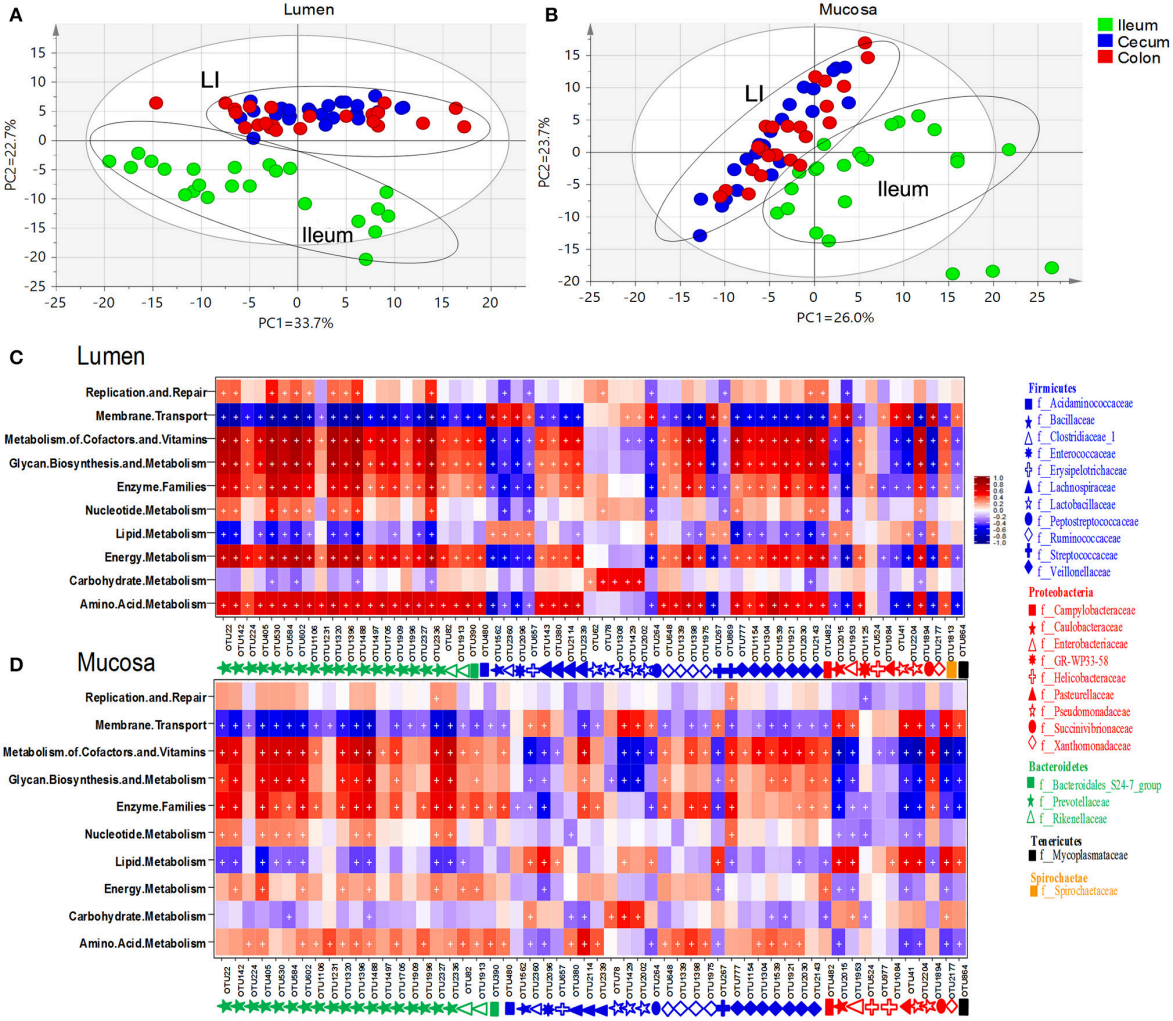
Principal component analyses (PCA) of gene functions of microbiome residing in lumen **(A)** and mucosa **(B)**. Heatmap illustrated correlations (red: positive; blue: negative) between phylogenetic groups at the level of genus and KEGG pathways for luminal microbiome **(C)** and mucosa-attached microbiome **(D)**. Significant correlations were indicated by “+” (*q* < 0.05).

To understand the functional differences among microbiome residing in distinct niches, 144 metagenomes were annotated with the KEGG pathway analyses to obtain a total of 39 categories of gene functions (Figure S2). Principal component analyses revealed a cluster of cecum and colon which was clearly separated from the ileum (Figures 7A,B). The most abundant gene functions included membrane transport (~13%), replication and repair (~9.59%), carbohydrate metabolism (~9.9%), amino acid metabolism (~8.6%), energy metabolism (~5.5%) and lipid metabolism (~2.79%) (Table S7). Of those six predominant gene functions, those involved in the metabolism of amino acid, energy and lipid were more enriched whereas membrane transport was lower in the ileal mucosa than those in the lumen. Gene functions involved in the metabolism of carbohydrate and lipid were overrepresented in the luminal microbiota of large intestine. In addition, genes related to lipid metabolism and amino acid metabolism were enriched in the lumen of cecum and colon, respectively (Table S7).

Similar patterns were found in the correlation between microbial gene functions and core intestinal OTU in the lumen (Figure 7C, Table S8) and mucosa (Figure 7D, Table S8). Core OTUs derived from *Bacteroidetes, Acidaminococcaceae, Lachnospiraceae, Ruminococcaceae* and *Veillonellaceae* within *Firmicutes*, and *Campylobacteraceae, GR-WP33-58, Succinivibrionaceae*, and *Spirochaetaceae* within *Proteobacteria* were positively correlated with gene functions related to metabolism of amino acid, energy and cofactors and vitamins, enzyme families, glycan biosynthesis and metabolism, but negatively correlated with lipid metabolism and membrane transport. An opposite pattern was found in the OTUs derived from *Bacillaceae, Clostridiaceae, Enterococcaceae, Erysipelotrichia, Peptostreptococcaceae, Streptococcaceae* within *Firmicutes, Caulobacteraceae, Enterobacteriaceae, Pseudomonadaceae, Xanthomonadaceae* within *Proteobacteria*, and *Mycoplasmataceae* within *Tenericutes*. Of note, the correlations were generally weaker in the mucosa-related microbiota compared with the luminal microbiota. However, OTUs of *Proteobacteria, Enterococcaceae, Streptococcaceae* found in mucosa communities showed a stronger positive correlation to lipid metabolism than their luminal counterparts.

## Discussion

Luminal and mucosa-attached microbiota in the swine intestine were dominated by *Firmicutes, Bacteroidetes* and *Proteobacteria*, similar to previous findings in pigs (Looft et al., 2014a; Mach et al., 2015; Sun et al., 2015, 2016; Umu et al., 2015; Kelly et al., 2016; Ramayocaldas et al., 2016) and humans (Qin et al., 2010). Significant differences in microbial richness, α-diversity and β-diversity were found among the three gut sections as well as between the lumen and mucosa, despite of the structural continuity of the investigated gut sections. Microbiome profile (abundance of OTU) as well as structure (presence or absence of OTU) contributed to the diversity of the microbial community residing in different gut niches. The increased *Prevotella* within *Bacteroidetes* and *Veillonellaceae, Lachnospiraceae* and *Ruminococcaceae* within the *Firmicutes* equip the large intestine with metabolic capabilities that are indispensable for host survival. Specifically, the taxonomic shifts in the *Prevotella* and genera of *Proteobacteria* (*Campylobacter, Helicobacter*, and *Lawsonia*) between the luminal and mucosal bacteria support the assumption that luminal microbiota may be more involved in the metabolism and digestion of nutrients, whereas the mucosal microbiome may be more involved in the immune function.

Along the length of the intestinal tract, great variations in *Bacteroietes, Firmicutes* and *Proteobacteria* were found between the ileum and the large intestine. Spatial changes in bacterial composition along the intestinal tract closely related to the dramatic changes in the intestinal microenvironments. First of all, oxygen availability significantly decreased from the ileum to the large intestine (He et al., 1999; Espey, 2013). Facultative anaerobes, such as *Enterobacteriaceae* (Donaldson et al., 2016) can easily grow in the ileum and anaerobic *Bacteroidetes* are adapted to low oxygen environment in the large intestine. Oxygen-sensitive *Bacteroidetes* remarkedly increased when oxygen-tolerant *Proteobacteria* decreased in the large intestine. Secondly, pH gradient along the intestine was another important factor to shape the dynamic changes of *Firmicutes and Bacteroides*. *Firmicutes* dominated at mildly acidic pH levels (~5.5) similar to the ileum environment, whereas *Bacteroides spp*. outcompeted them at close to neutral pH levels (~6.7) (Duncan et al., 2009). Thirdly, most of dietary nutrients are fully digested at the end of ileum under normal physiological conditions, and undigested complex carbohydrates accumulate in the large intestine and undergo bacterial fermentation. In growing pigs, the apparent ileal digestibility of carbohydrates is about 70% (Chen et al., 2013), which means approximately 30% carbohydrates enter the lower intestinal sections. Therefore, microbiota capable of degrading complex carbohydrates, including *Bacteroidetes* (Arumugam et al., 2011) and some families of *Firmicutes* (*Veillonellaceae, Lachnospiraceae*, and *Ruminococcaceae*) (Rode et al., 1981; Duncan et al., 2007), were significantly increased in the large intestine. Microbial fermentation capabilities are greatly promoted, indicated by the over 5-fold increase in the SCFA production.

In the current study, we showed a great difference in total bile acid pool in different gut sections, which was reduced by 1/3 in the large intestine compared with the ileum. Bile salts are known to be important for bacteria colonization. In the ileum, the number of bile salt-sensitive bacteria is limited. For example, *Prevotellaceae*, highly sensitive to bile salts (Krause and Russell, 1996), made of <10% of ileal microbiota, but became the dominant bacterial family in the cecum and colon. Redundancy analyses further showed that the ileal microbiota positively correlated with primary and conjugated bile acids but negatively correlated to secondary bile acids, whereas the microbiota communities in the cecum and colon had an opposite relationship with bile acids. Interactions have been widely reported for bile salts and intestinal microbiota (Kurdi et al., 2006; Wahlström et al., 2016). Many ileal-dominant microbiota, such as *Lactobacillus* and *Clostridium* (Archer et al., 1982), have bile acid inducible genes (BSH activities) to deconjugate bile acids and convert primary bile acids to secondary bile acids, a process important for bile acids metabolism. It is believed that the composition of intestinal bile acid pool is one of critical driven forces in shaping topographical distribution of microbiota in the intestine.

Intestinal microbiome is capable of various metabolic functions that are lacking in the host, and therefore is important for the life of host animals. Our results showed *Bacteroidetes, Veillonellaceae, Lachnospiraceae*, and *Ruminococcaceae* were positively correlated with metabolism of amino acid, energy, cofactors and vitamins, secondary metabolites and glycan. Since these taxa dominated in the large intestine, microbial metabolic activities in the large intestine are very important routes for digestion of complex carbohydrates and biotransformation of amino acids and vitamins in the hosts (Gill et al., 2006).

Mucosal microbiota that colonize in the mucus layer have a notable role in immunomodulation and gut-brain communication (Bienenstock et al., 2015; Min and Rhee, 2015). The physicochemical conditions and substrate availability of mucosa and lumen create diversified microenvironments that support diverse microbial populations (Stearns et al., 2011). The spatial distribution of mucosa-associated microbiota differed from that of the luminal microbiota, where mucosal *Bacteroidetes* significantly increased when *Proteobacteria* rather than *Firmicutes* decreased in the cecum and colon. In the same segment of cecum and colon, enrichment in *Campylobacter, Helicobacter, Pseudomonas*, and *Lawsonia*, but lower abundance of *Prevotella_9 and Prevotella_2* were found in the mucosa in comparison to the lumen. This observation is consistent with the study conducted in weaning piglets by Kelly et al. (2016), who found microaerophilic *Helicobacteraceae* and *Campylobacteraceae* were enriched in the mucosa, whereas obligate anaerobic bacteria from *Prevotellaceae, Lachnospiraceae, Ruminococcaceae*, and *Veillonellaceae* were abundant in the lumen of the cecum (Kelly et al., 2016). Oxygen diffusion from the epithelial capillary network creates an oxygen-abundant microenvironment in the mucosa (Albenberg et al., 2014). The oxygen gradient at the radius level has great impacts on the differential colonization of bacteria in the lumen and mucosa. In addition, *Campylobacter* and *Helicobacter* have rapid motility at high viscosity (Beeby, 2015), and great mucin-colonizing ability (Naughton et al., 2013), making them adept at the outer mucus layer. More importantly, *Campylobacter* (Lastovica et al., 2014), *Helicobacter* (Cahill et al., 1997), and *Lawsonia* (McCluskey et al., 2002) are obligate intracellular bacteria. The commensal presence of those pathogens potentially stimulates the immunoprotecting function of the intestinal epithelial barrier. However, overgrowth of these microbes disturbs the intestinal barrier function and consequently results in enteric diseases.

Distinct differences were revealed in the co-occurrence of core microbiota in the lumen and mucosa of pigs. *Prevotellaceae, Ruminococcaceae, Lachnospiraceae* and *Veillonellaceae* very likely assemble a functional group in the lumen of healthy piglets, which centers the microbial network in the lumen. *Prevotella* and *Ruminococcus* were also identified as central genera of the two enterotype-like clusters related to growth traits of commercial pigs (Ramayocaldas et al., 2016). Cross-feeding/co-metabolism occurs between *Prevotella* and *Ruminococcaceae, Lachnospiraceae* and *Veillonellaceae* to utilize mucin to produce butyrate (Wright et al., 2000; Fischbach and Sonnenburg, 2011). All these data suggested that microbial capabilities to degrade complex carbohydrates and produce SCFAs are of great importance to the growth performance of swine. The co-abundance of functionally-similar organisms in the same niche is considered to be an important feature of the gut ecosystem to maintain the robustness and resilience (Moya and Ferrer, 2016). In the mucosa, *Prevotellaceae* was co-abundant with other SCFA producers as their luminal counterparts. In addition, co-abundance of opportunistic pathogens from γ-*Proteobacteria* assemble the other function microbial group in the mucosa. All taxa of γ-*Proteobacteria* are Gram-negative with a lipopolysaccharide-containing outer membrane, which can elicit proinflammatory effects in susceptible hosts. An expansion of *Proteobacteria* was noted in the inflammatory bowel disease (IBD) (Mukhopadhya et al., 2012), suggesting the co-occurrence of mucosa-association is closely related to the occurrence of inflammation.

Noteworthy, the *Enterobacteriaceae* of *Proteobacteria* was co-exclusive to *Prevotellaceae*-centered microbial groups in the lumen. Negative interactions between *Enterobacteriaceae* and *Prevotellaceae, Ruminococcaceae*, and *Lachnospiraceae* were also observed in several diseases, such as Parkinson Disease (Scheperjans et al., 2015), IBD (Morgan et al., 2012; Mukhopadhya et al., 2012), hepatic encephalophy (Bajaj, 2014), and enteritis (Mon et al., 2015). The co-exclusion relationship between *Enterobacteriaceae* and *Prevotellaceae*-centered microbial groups are particularly important for the health of the intestine. Bacteria belonging to *Bacteroidia* (*Provotellaceae*) and *Clostridia* (*Ruminococcaceae, Lachnospiraceae*) have great capacities in the complex carbohydrates digestion to produce SCFAs. SCFAs can directly reduce the growth of *Enterobacteriaceae* by lowing the pH. They also can reduce the colonization of *Enterobacteriaceae* by suppressing inflammations. Facultative anaerobic *Proteobacteria*, including *Enterobacteriaceae*, are able to respire nitrate or other electron acceptors generated by inflammations (Winter et al., 2010, 2013). In contrast, obligate anaerobic *Clostridia* and *Bacteroidia* lack the terminal oxidoreductases needed to use those exogenous electron acceptors. Via GPR43, SCFAs interact with T_reg_ cells to increase IL-10 and limit effector CD4^+^ T cells to reduce the inflammatory responses (Atarashi et al., 2011; Smith et al., 2013), consequently decreasing *Enterobacteriaceae* colonization. The colonization resistance between obligate anaerobic *Bacteroidia* and *Clostridia* and facultative anaerobic *Enterobacteriaceae* is one of the mechanisms to prevent intestinal infections (Spees et al., 2013).

**Figure 8:**
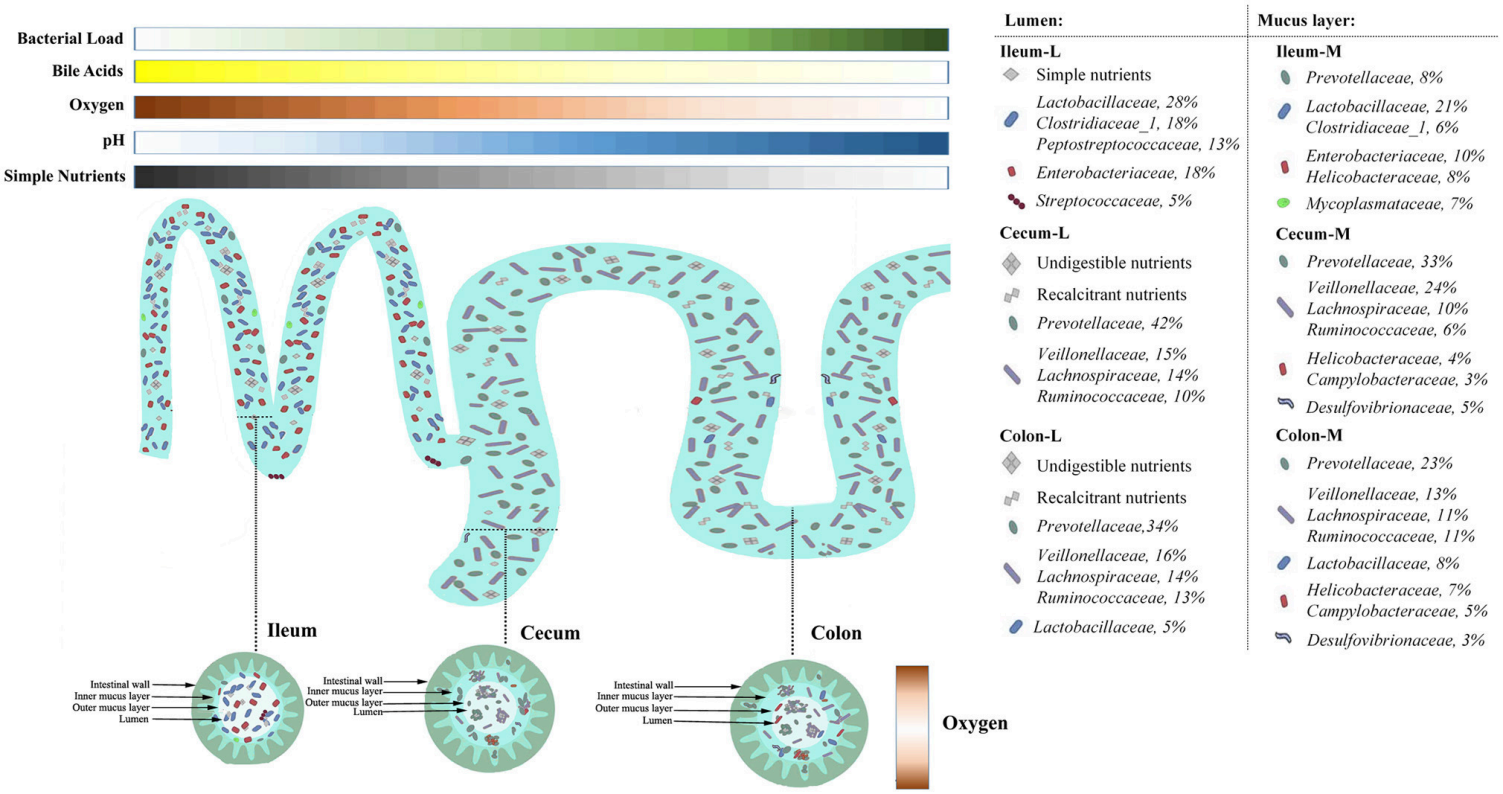
Spatial heterogeneity of microbial communities in different niches of swine intestine. Nutrients availability, gradients of pH, oxygen as well as bile acids along the length of the intestine shape the size and structure of microbial community in the ileum and the large intestine, respectively. In the ileum, abundant simple nutrients, mildly acidic pH, microaerobic environment sustain the growth of bile acid-tolerant, facultative taxa. In contrast, the large intestine is populated by anaerobic taxa that can utilize complex carbohydrates. Heterogeneity in microbial assembly, structure and function is present along the radical axis when nutrition sources and oxygen sustain the growth of some opportunistic pathogens in the mucosa, such as *Camypylobacteraceae* and *Helicobacteraceae*.

By taking advantages of next generation sequencing, the current study obtains a topographical map of swine gut microbiome. Heterogeneities in microbial assembly, structure and function are noted in six compartments of swine intestine, representing different natural niches. Co-occurrence network analyses reveal two potential functional microbial groups in the lumen and mucosa. The *Prevotellaceae*-centered group is greatly involved in the utilization of refractory carbohydrates to produce SCFAs, while the *Proteobacteria* group is of importance in the immune response. Putting our findings and previous data together, it showed substrate availability, oxygen gradients, pH as well as SCFAs and bile acids and co-existence of microorganisms are critical gut microenvironmental factors shaping the microbial communities residing in the swine intestine (Figure 8). This work will greatly facilitate development of strategies for targeting gut microbiome manipulation to improve the health and production efficiency of pigs.

## Author contributions

Conceived and designed the experiments: JX, HZ, LZ, and WW. Performed the experiments: LZ and WW. Analyzed the data: LZ. Manuscript preparation: LZ and JX. Manuscript revisions: JX and Y-KL.

### Conflict of interest statement

The authors declare that the research was conducted in the absence of any commercial or financial relationships that could be construed as a potential conflict of interest.

## References

[B1] Albenberg L. Esipova T. V. Judge C. P. Bittinger K. Chen J. Laughlin A.. (2014). Correlation between intraluminal oxygen gradient and radial partitioning of intestinal microbiota. Gastroenterology 147, 1055–63.e8. 10.1053/j.gastro.2014.07.020 25046162 PMC4252572

[B2] Archer R. Chong R. Maddox I. (1982). Hydrolysis of bile acid conjugates by *Clostridium bifermentans*. Appl. Microbiol. Biotechnol. 14, 41–45. 10.1007/BF00508002

[B3] Arumugam M. Raes J. Pelletier E. Le Paslier D. Yamada T. Mende D. R.. (2011). Enterotypes of the human gut microbiome. Nature 473, 174–180. 10.1038/nature09944 21508958 PMC3728647

[B4] Atarashi K. Tanoue T. Shima T. Imaoka A. Kuwahara T. Momose Y.. (2011). Induction of colonic regulatory T cells by indigenous Clostridium species. Science 331, 337–341. 10.1126/science.1198469 21205640 PMC3969237

[B5] Bajaj J. S. (2014). The role of microbiota in hepatic encephalopathy. Gut Microbes 5, 397–403. 10.4161/gmic.28684 24690956 PMC4153779

[B6] Beeby M. (2015). Motility in the epsilon-proteobacteria. Curr. Opin. Microbiol. 28, 115–121. 10.1016/j.mib.2015.09.005 26590774

[B7] Bienenstock J. Kunze W. Forsythe P. (2015). Microbiota and the gut–brain axis. Nutr. Rev. 73, 28–31. 10.1093/nutrit/nuv019 26175487

[B8] Bokulich N. A. Subramanian S. Faith J. J. Gevers D. Gordon J. I. Knight R.. (2013). Quality-filtering vastly improves diversity estimates from Illumina amplicon sequencing. Nat. Methods 10, 57–59. 10.1038/nmeth.2276 23202435 PMC3531572

[B9] Cahill R. J. Foltz C. J. Fox J. G. Dangler C. A. Powrie F. Schauer D. B. (1997). Inflammatory bowel disease: an immunity-mediated condition triggered by bacterial infection with Helicobacter hepaticus. Infect. Immun. 65, 3126–3131. 9234764 10.1128/iai.65.8.3126-3131.1997 PMC175441

[B10] Caporaso J. G. Lauber C. L. Walters W. A. Berg-Lyons D. Huntley J. Fierer N.. (2012). Ultra-high-throughput microbial community analysis on the Illumina HiSeq and MiSeq platforms. ISME J. 6, 1621–1624. 10.1038/ismej.2012.8 22402401 PMC3400413

[B11] Carding S. Verbeke K. Vipond D. T. Corfe B. M. Owen L. J. (2015). Dysbiosis of the gut microbiota in disease. Microb. Ecol. Health Dis. 26:26191. 10.3402/mehd.v26.26191 25651997 PMC4315779

[B12] Chen L. Zhang H. Gao L. Zhao F. Lu Q. Sa R. (2013). Effect of graded levels of fiber from alfalfa meal on intestinal nutrient and energy flow, and hindgut fermentation in growing pigs. J. Anim. Sci. 91, 4757–4764. 10.2527/jas.2013-6307 23965393

[B13] Clarke K. R. (1993). Non-parametric multivariate analyses of changes in community structure. Austral Ecol. 18, 117–143. 10.1111/j.1442-9993.1993.tb00438.x

[B14] Donaldson G. P. Lee S. M. Mazmanian S. K. (2016). Gut biogeography of the bacterial microbiota. Nat. Rev. Microbiol. 14, 20–32. 10.1038/nrmicro3552 26499895 PMC4837114

[B15] Duncan S. H. Louis P. Thomson J. M. Flint H. J. (2009). The role of pH in determining the species composition of the human colonic microbiota. Environ. Microbiol. 11, 2112–2122. 10.1111/j.1462-2920.2009.01931.x 19397676

[B16] Duncan S. Louis P. Flint H. (2007). Cultivable bacterial diversity from the human colon. Lett. Appl. Microbiol. 44, 343–350. 10.1111/j.1472-765X.2007.02129.x 17397470

[B17] Edgar R. C. (2013). UPARSE: highly accurate OTU sequences from microbial amplicon reads. Nat. Methods 10, 996–998. 10.1038/nmeth.2604 23955772

[B18] Edgar R. C. Haas B. J. Clemente J. C. Quince C. Knight R. (2011). UCHIME improves sensitivity and speed of chimera detection. Bioinformatics 27, 2194–2200. 10.1093/bioinformatics/btr381 21700674 PMC3150044

[B19] Espey M. G. (2013). Role of oxygen gradients in shaping redox relationships between the human intestine and its microbiota. Free Radic. Biol. Med. 55, 130–140. 10.1016/j.freeradbiomed.2012.10.554 23127782

[B20] Fallingborg J. (1999). Intraluminal pH of the human gastrointestinal tract. Dan. Med. Bull. 46, 183–196 10421978

[B21] Faust K. Sathirapongsasuti J. F. Izard J. Segata N. Gevers D. Raes J.. (2012). Microbial co-occurrence relationships in the human microbiome. PLoS Comput. Biol. 8:e1002606. 10.1371/journal.pcbi.1002606 22807668 PMC3395616

[B22] Fischbach M. A. Sonnenburg J. L. (2011). Eating for two: how metabolism establishes interspecies interactions in the gut. Cell Host Microbe 10, 336–347. 10.1016/j.chom.2011.10.002 22018234 PMC3225337

[B23] Friedman J. Alm E. J. (2012). Inferring correlation networks from genomic survey data. PLoS Comput. Biol. 8:e1002687. 10.1371/journal.pcbi.1002687 23028285 PMC3447976

[B24] Gill S. R. Pop M. DeBoy R. T. Eckburg P. B. Turnbaugh P. J. Samuel B. S.. (2006). Metagenomic analysis of the human distal gut microbiome. Science 312, 1355–1359. 10.1126/science.1124234 16741115 PMC3027896

[B25] He G. Shankar R. A. Chzhan M. Samouilov A. Kuppusamy P. Zweier J. L. (1999). Noninvasive measurement of anatomic structure and intraluminal oxygenation in the gastrointestinal tract of living mice with spatial and spectral EPR imaging. Proc. Natl. Acad. Sci. U.S.A. 96, 4586–4591. 10.1073/pnas.96.8.4586 10200306 PMC16376

[B26] Heinsen F. A. Knecht H. Neulinger S. C. Schmitz R. A. Knecht C. Kühbacher T.. (2015). Dynamic changes of the luminal and mucosa-associated gut microbiota during and after antibiotic therapy with paromomycin. Gut Microbes 6, 243–254. 10.1080/19490976.2015.1062959 26178862 PMC4615565

[B27] Hollister E. B. Gao C. Versalovic J. (2014). Compositional and functional features of the gastrointestinal microbiome and their effects on human health. Gastroenterology 146, 1449–1458. 10.1053/j.gastro.2014.01.052 24486050 PMC4181834

[B28] Kelly J. Daly K. Moran A. W. Ryan S. Bravo D. Shirazi-Beechey S. P. (2016). Composition and diversity of mucosa-associated microbiota along the entire length of the pig gastrointestinal tract; dietary influences. Environ. Microbiol. 19, 1425–1438. 10.1111/1462-2920.13619 27871148

[B29] Krause D. Russell J. (1996). How many ruminal bacteria are there? J. Dairy Sci. 79, 1467–1475. 10.3168/jds.S0022-0302(96)76506-2 8880472

[B30] Kurdi P. Kawanishi K. Mizutani K. Yokota A. (2006). Mechanism of growth inhibition by free bile acids in lactobacilli and bifidobacteria. J. Bacteriol. 188, 1979–1986. 10.1128/JB.188.5.1979-1986.2006 16484210 PMC1426545

[B31] Langille M. G. Zaneveld J. Caporaso J. G. McDonald D. Knights D. Reyes J. A.. (2013). Predictive functional profiling of microbial communities using 16S rRNA marker gene sequences. Nat. Biotechnol. 31, 814–821. 10.1038/nbt.2676 23975157 PMC3819121

[B32] Lastovica A. J. On S. L. W. Li Z. (2014). The Family Campylobacteraceae. Berlin; Heidelberg: Springer

[B33] Li H. Limenitakis J. P. Fuhrer T. Geuking M. B. Lawson M. A. Wyss M.. (2015). The outer mucus layer hosts a distinct intestinal microbial niche. Nat. Commun. 6:8292. 10.1038/ncomms9292 26392213 PMC4595636

[B34] Looft T. Allen H. K. Cantarel B. L. Levine U. Y. Bayles D. O. Alt D. P.. (2014a). Bacteria, phages and pigs: the effects of in-feed antibiotics on the microbiome at different gut locations. ISME J. 8, 1566–1576. 10.1038/ismej.2014.12 24522263 PMC4817603

[B35] Looft T. Allen H. K. Casey T. A. Alt D. P. Stanton T. B. (2014b). Carbadox has both temporary and lasting effects on the swine gut microbiota. Front. Microbiol. 5:276. 10.3389/fmicb.2014.00276 24959163 PMC4050737

[B36] Lozupone C. Knight R. (2005). UniFrac: a new phylogenetic method for comparing microbial communities. Appl. Environ. Microbiol. 71, 8228–8235. 10.1128/AEM.71.12.8228-8235.2005 16332807 PMC1317376

[B37] Mach N. Berri M. Estelle J. Levenez F. Lemonnier G. Denis C.. (2015). Early-life establishment of the swine gut microbiome and impact on host phenotypes. Environ. Microbiol. Rep. 7, 554–569. 10.1111/1758-2229.12285 25727666

[B38] Maidak B. L. Cole J. R. Lilburn T. G. Parker C. T. Jr. Saxman P. R. Farris R. J.. (2001). The RDP-II (ribosomal database project). Nucleic Acids Res. 29, 173–174. 10.1093/nar/29.1.173 11125082 PMC29785

[B39] McCluskey J. Hannigan J. Harris J. D. Wren B. Smith D. G. (2002). LsaA, an antigen involved in cell attachment and invasion, is expressed by *Lawsonia intracellularis* during infection *in vitro* and *in vivo*. Infect. Immun. 70, 2899–2907. 10.1128/IAI.70.6.2899-2907.2002 12010978 PMC128020

[B40] McMurdie P. J. Holmes S. (2013). phyloseq: an R package for reproducible interactive analysis and graphics of microbiome census data. PLoS ONE 8:e61217. 10.1371/journal.pone.0061217 23630581 PMC3632530

[B41] Min Y. W. Rhee P. L. (2015). The role of microbiota on the gut immunology. Clin. Ther. 37, 968–975. 10.1016/j.clinthera.2015.03.009 25846321

[B42] Mon K. K. Saelao P. Halstead M. M. Chanthavixay G. Chang H. C. Garas L.. (2015). Salmonella enterica serovars enteritidis infection alters the indigenous microbiota diversity in young layer chicks. Front. Vet. sci. 2:61. 10.3389/fvets.2015.00061 26664988 PMC4672283

[B43] Morgan X. C. Tickle T. L. Sokol H. Gevers D. Devaney K. L. Ward D. V.. (2012). Dysfunction of the intestinal microbiome in inflammatory bowel disease and treatment. Genome Biol. 13:R79. 10.1186/gb-2012-13-9-r79 23013615 PMC3506950

[B44] Moya A. Ferrer M. (2016). Functional redundancy-induced stability of gut microbiota subjected to disturbance. Trends Microbiol. 24, 402–413. 10.1016/j.tim.2016.02.002 26996765

[B45] Mukhopadhya I. Hansen R. El-Omar E. M. Hold G. L. (2012). IBD—what role do Proteobacteria play? Nat. Rev. Gastroenterol. Hepatol. 9, 219–230. 10.1038/nrgastro.2012.14 22349170

[B46] Naughton J. A. Mariño K. Dolan B. Reid C. Gough R. Gallagher M. E.. (2013). Divergent mechanisms of interaction of *Helicobacter pylori* and *Campylobacter jejuni* with mucus and mucins. Infect. Immun. 81, 2838–2850. 10.1128/IAI.00415-13. 23716616 PMC3719574

[B47] Paul D. Kumbhare S. V. Mhatre S. S. Chowdhury S. P. Shetty S. A. Marathe N. P.. (2015). Exploration of microbial diversity and community structure of lonar lake: the only hypersaline meteorite crater lake within basalt rock. Front. Microbiol. 6:1553. 10.3389/fmicb.2015.01553 26834712 PMC4722114

[B48] Pereira F. C. Berry D. (2017). Microbial nutrient niches in the gut. Environ. Microbiol. 19, 1366–1378. 10.1111/1462-2920.13659 28035742 PMC5412925

[B49] Qin J. Li R. Raes J. Arumugam M. Burgdorf K. S. Manichanh C.. (2010). A human gut microbial gene catalogue established by metagenomic sequencing. Nature 464, 59–65. 10.1038/nature08821 20203603 PMC3779803

[B50] Ramayocaldas Y. Mach N. Lepage P. Levenez F. Denis C. Lemonnier G. (2016). Phylogenetic network analysis applied to pig gut microbiota identifies an ecosystem structure linked with growth traits. ISME J. 10, 2973–2977. 10.1038/ismej.2016.77 27177190 PMC5148198

[B51] Rastall R. A. (2004). Bacteria in the gut: friends and foes and how to alter the balance. J. Nutr. 134(8 Suppl.), 2022S–2026S. 15284393 10.1093/jn/134.8.2022S

[B52] Ridlon J. M. Kang D. J. Hylemon P. B. Bajaj J. S. (2014). Bile Acids and the Gut Microbiome. Curr. Opin. Gastroenterol. 30, 332–338. 10.1097/MOG.0000000000000057 24625896 PMC4215539

[B53] Rode L. Genthner B. S. Bryant M. (1981). Syntrophic association by cocultures of the methanol-and CO2-H2-utilizing species Eubacterium limosum and pectin-fermenting Lachnospira multiparus during growth in a pectin medium. Appl. Environ. Microbiol. 42, 20–22. 16345811 10.1128/aem.42.1.20-22.1981 PMC243954

[B54] Scheperjans F. Aho V. Pereira P. A. Koskinen K. Paulin L. Pekkonen E.. (2015). Gut microbiota are related to Parkinson's disease and clinical phenotype. Mov. Dis. 30, 350–358. 10.1002/mds.26069 25476529

[B55] Smith P. M. Howitt M. R. Panikov N. Michaud M. Gallini C. A. Bohlooly Y. M.. (2013). The microbial metabolites, short-chain fatty acids, regulate colonic Treg cell homeostasis. Science 341, 569–573. 10.1126/science.1241165 23828891 PMC3807819

[B56] Spees A. M. Lopez C. A. Kingsbury D. D. Winter S. E. (2013). Colonization resistance: battle of the bugs or Menage a Trois with the host? PLoS Pathog. 9:e1003730 10.1371/journal.ppat.1003730 24278012 PMC3836981

[B57] Stearns J. C. Lynch M. D. Senadheera D. B. Tenenbaum H. C. Goldberg M. B. Cvitkovitch D. G.. (2011). Bacterial biogeography of the human digestive tract. Sci. Rep. 1:170. 10.1038/srep00170 22355685 PMC3240969

[B58] Sun Y. Su Y. Zhu W. (2016). Microbiome-metabolome responses in the cecum and colon of pig to a high resistant starch diet. Front. Microbiol. 7:779. 10.3389/fmicb.2016.00779 27303373 PMC4880592

[B59] Sun Y. Zhou L. Fang L. Su Y. Zhu W. (2015). Responses in colonic microbial community and gene expression of pigs to a long-term high resistant starch diet. Front. Microbiol. 6:877. 10.3389/fmicb.2015.00877 26379652 PMC4548152

[B60] Umu Ö. C. O. Frank J. A. Fangel J. U. Oostindjer M. Silva C. S. D. Bolhuis E. J.. (2015). Resistant starch diet induces change in the swine microbiome and a predominance of beneficial bacterial populations. Microbiome 3, 1–15. 10.1186/s40168-015-0078-5 25905018 PMC4405844

[B61] Wahlström A. Sayin S. I. Marschall H. U. Bäckhed F. (2016). Intestinal crosstalk between bile acids and microbiota and its impact on host metabolism. Cell Metab. 24, 41–50. 10.1016/j.cmet.2016.05.005 27320064

[B62] Walter J. Ley R. (2011). The human gut microbiome: ecology and recent evolutionary changes. Annu. Rev. Microbiol. 65, 411–429. 10.1146/annurev-micro-090110-102830 21682646

[B63] Winter S. E. Thiennimitr P. Winter M. G. Butler B. P. Huseby D. L. Crawford R. W.. (2010). Gut inflammation provides a respiratory electron acceptor for Salmonella. Nature 467, 426–429. 10.1038/nature09415 20864996 PMC2946174

[B64] Winter S. E. Winter M. G. Xavier M. N. Thiennimitr P. Poon V. Keestra A. M.. (2013). Host-derived nitrate boosts growth of *E. coli* in the inflamed gut. Science 339, 708–711. 10.1126/science.1232467 23393266 PMC4004111

[B65] Wright D. P. Rosendale D. I. Roberton A. M. (2000). Prevotella enzymes involved in mucin oligosaccharide degradation and evidence for a small operon of genes expressed during growth on mucin. FEMS Microbiol. Lett. 190, 73–79. 10.1111/j.1574-6968.2000.tb09265.x 10981693

[B66] Wu W. Xie J. Zhang H. (2016). Dietary fibers influence the intestinal SCFAs and plasma metabolites profiling in growing pigs. Food Funct. 7, 4644–4654. 10.1039/C6FO01406B 27754504

[B67] Yang H. Huang X. Fang S. Xin W. Huang L. Chen C. (2016). Uncovering the composition of microbial community structure and metagenomics among three gut locations in pigs with distinct fatness. Sci. Rep. 6:27427. 10.1038/srep27427 27255518 PMC4891666

[B68] Zhao W. Wang Y. Liu S. Huang J. Zhai Z. He C.. (2015). The dynamic distribution of porcine microbiota across different ages and gastrointestinal tract segments. PLoS ONE 10:e0117441. 10.1371/journal.pone.0117441 25688558 PMC4331431

