# Supplementary material for: Spatial Heterogeneity and Co-occurrence of Mucosal and Luminal Microbiome across Swine Intestinal Tract
Source: Front Microbiol. 2018 Jan 26;9:48. doi: 10.3389/fmicb.2018.00048 (PMC5810300; doi:10.3389/fmicb.2018.00048)
Supplement: Supplementary file 9 [file Presentation1.PPTX]

## Slide 1
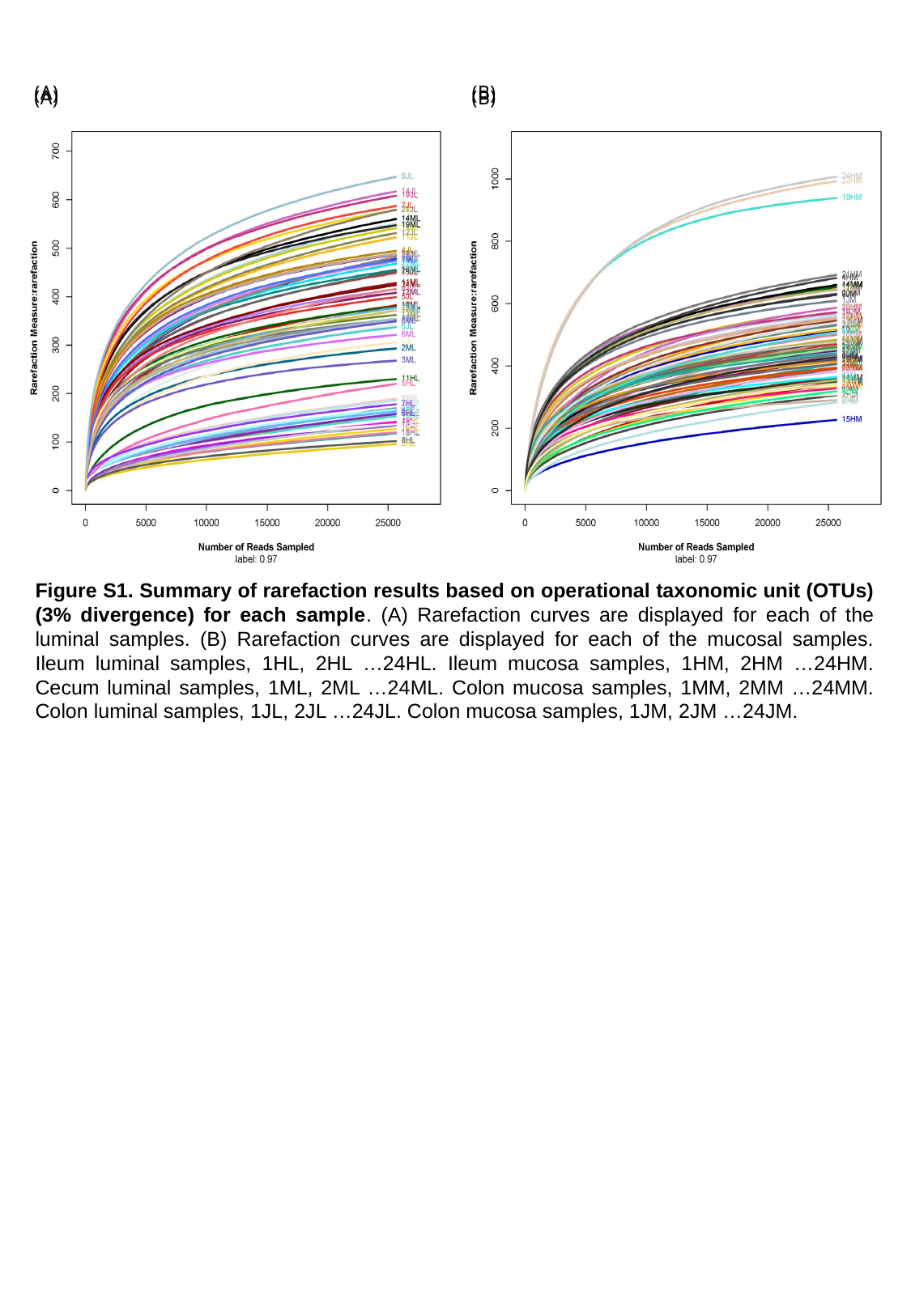

Figure S1. Summary of rarefaction results based on operational taxonomic unit (OTUs) (3% divergence) for each sample. (A) Rarefaction curves are displayed for each of the luminal samples. (B) Rarefaction curves are displayed for each of the mucosal samples. Ileum luminal samples, 1HL, 2HL …24HL. Ileum mucosa samples, 1HM, 2HM …24HM. Cecum luminal samples, 1ML, 2ML …24ML. Colon mucosa samples, 1MM, 2MM …24MM. Colon luminal samples, 1JL, 2JL …24JL. Colon mucosa samples, 1JM, 2JM …24JM.

## Slide 2
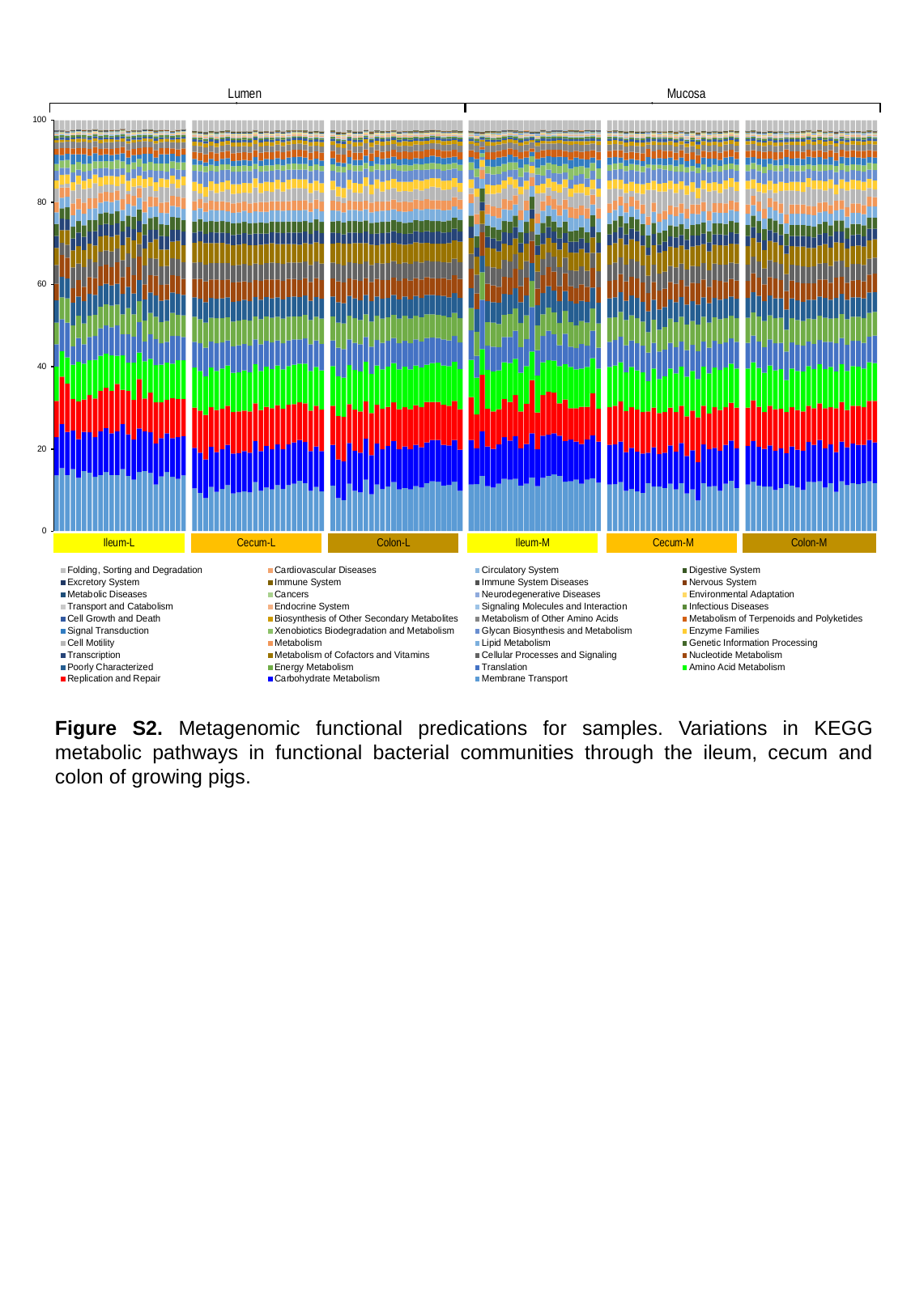

Figure S2. Metagenomic functional predications for samples. Variations in KEGG metabolic pathways in functional bacterial communities through the ileum, cecum and colon of growing pigs.
